# Early calf slaughter: impact of industry-led policy interventions on trends in Ireland, 2024

**DOI:** 10.3389/fvets.2025.1629858

**Published:** 2025-11-26

**Authors:** Sinnead Oakes, Eoin Ryan, Andrew W. Byrne

**Affiliations:** Department of Agriculture, Food and the Marine, Government of Ireland, Dublin, Ireland

**Keywords:** calf slaughter, dairy, male calf, welfare, tuberculosis

## Abstract

The dairy industry produces a surplus of male calves with low monetary value and slaughtering them at a young age has been used as a means of disposal, raising ethical concerns. In 2024, changes to the dairy industry quality assurance standards were introduced to prevent this practice in Ireland. The objectives of the present study were to explore trends in calf slaughter, measure the effects of the intervention, and to identify any unintended consequences of the industry-led policy. The data on 16,598 <56-day-old calves slaughtered from a total of 1,937,533 born, January to May 2024, were obtained from national databases. We fitted negative binomial regression models to the count of slaughtered calves per birth herd to assess associated contributory factors. The study revealed that there has been a drop in early calf slaughter from an average of 1.09% of calves in 2018–2022 to 0.86% in 2024. There were 1,241 birth herds of slaughtered calves but only 247 herds presented calves for slaughter. Furthermore, 1,019 of the birth herds moved calves to a presenting herd before slaughter. The birth herds were mostly members of the dairy industry quality assurance scheme (97%). The presenting herds had lower membership (83%) and tended to send larger number of calves. One single presenting herd accounted for 20% of the total slaughtered calves. These results show that the intervention had a sizable effect in its first year and highlights the potential for industry-driven policy changes to influence the choices farmers make, and the need for continual monitoring.

## 1 Introduction

Disposing of male dairy calves as an industry by-product through slaughter is a contentious practice raising socio-ethical issues among stakeholders and the general public (1, 2), and has led to significant debate and condemnation of the source production systems [e.g., “bobby calves” slaughtered at 4–7 days in New Zealand (3)]. Additionally, it creates a reputational challenge for the dairy industry, can represent a sizable economic cost (4, 5) and has been shown to be a potential indicator of animal welfare issues on-farm (6). The almost singular potential utility of the dairy sired, male dairy calf is in beef production. In Ireland, the commercial beef value (CBV) is a Euro-based metric developed by industry stakeholders to forecast the economic value of a bovine as a beef finished animal by scoring it on a host of genetic traits relevant to beef production. Dairy calves have the lowest CBV and therefore the least economic value. Indeed the 2024 published figures quoted the average CBVs for a Beef sire, Beef dam bred calf as €241 whereas the Dairy sire, Dairy dam calf was €-1 (7).

A recent Irish study revealed a year-on-year increase in the number of male dairy calves slaughtered during the period of the study 2018–2022 (8). It was observed that a small number of farms were slaughtering a high number of calves, and on a more consistent basis, and that these were among the larger sized and more recently established herds. This pattern suggested a possible correlation between the abolition of dairy quotas (in 2015), the consequent increase in herd size and the observed increased calf slaughter rates.

Bord Bia is an Irish semi-state agency mandated with promoting Irish food produce nationally and internationally. In 2014, a technical advisory committee representing key Irish dairy industry stakeholders including Bord Bia developed the Sustainable Dairy Assurance Scheme (SDAS), open to all bovine milk producers with an Irish herd number (9). The goal of the scheme was to enhance consumer confidence in a sustainable, ethical and quality-assured dairy industry. Members of the scheme must achieve a prescribed standard across a host of criteria and are subsequently awarded certification and a slew of economic benefits including target market access opportunities and a higher market value for their cattle.

In response to concerns in the industry regarding the number of male dairy calves slaughtered at an early age, the standard was revised such that members were not permitted to send healthy calves to slaughter before the age of 8 weeks, other than in cases of an animal disease outbreak such as bovine tuberculosis (bTB) or other *force majeure*. Also explicitly prohibited is any intentional movement of calves off farm for the purpose of early slaughter. Members of the scheme may be audited by the quality assurance body and if found to be contravening the new rules could face possible loss of their membership of the SDAS. Such a loss would likely incur financial losses as most milk processors prefer to only buy milk from quality assurance scheme members. These new criteria came into effect on January 1st 2024. The purpose of the present study was to examine the trends in calf slaughter data pre- and post- this industry-led policy and to observe the impact and outcomes of these interventions.

## 2 Methods

### 2.1 Data collection

This study extends a previous analysis of the trends and factors associated with dairy calf early slaughter in Ireland, 2018–2022 (8). The description “male dairy calf” refers to the male progeny of a dairy type dam and sire. We defined early calf slaughter (ECS) as the slaughter of healthy calves before 8 weeks (56 days) of age, for the purpose of disposal. As there are no veal production systems in Ireland, we assumed that, except for reasons of *force majeure* such as disease outbreak in the herd, all calves slaughtered are ECS.

In the current study the data on ECS was collected between January 2024 and May 2024 to coincide with the peak calving period. Calf movements were obtained from the Animal Identification and Movement System (AIMS) within the Department of Agriculture, Food and the Marine. Based on the aforementioned previous study the variables selected as significant included calf breed type, sex of the calf, whether or not the birth herd or herd of presentation had membership of the SDAS, whether or not the calf had moved from the birth herd (BH) to a second herd prior to presentation (presenting herd, PH) for slaughter and finally the bTB status of the birth herd. Herd size of the birth herd was also explored as a predictor of ECS (8). Breed and breed type were as reported by the herd owner.

### 2.2 Data analysis

The data was analyzed using MS Excel and Stata [Stata MP version 16; (10)]. The trends in movement and slaughter patterns between dairy and beef farms which were, and were not, SDAS members were explored descriptively as were the difference in numbers of male versus female calves in each of the categories. The bTB status of the birth herd was also considered as a previous study found a small increase in calf slaughter rates in herds experiencing a bTB breakdown (8).

Following (8), we fitted negative binomial regression models to the count of slaughtered calves per birth herd to assess factors contributing to higher ECS. Negative binomial models were used as the variance was larger than the mean count, and a likelihood ratio test of the overdispersion parameter suggested the model fitted the data better than a Poisson equivalent model. Candidate predictor variables included bTB status (2024), herd size, breed type (3-way categorical, majority Friesian/Friesian X, majority Jersey/Jersey X, other), and whether the herd was Bord Bia certified. All variables were fitted to the model as there was *a priori* evidence of their association with calf slaughter and were significantly associated in the multivariable model (*α*: *p* < 0.05). A directed acyclic graph (DAG) is presented in Supplementary Figure S2 to illustrate the relationship between variables. Exponentiated coefficients (Incidence Rate Ratios; IRRs) are reported throughout, which represent the multiplicative change in the expected count for a one-unit increase in a predictor.

## 3 Results

There were 1,937,533 calves registered as born between January and May 2024, of which approximately 90% were retained locally in either the dairy or beef industry. During this period 114,596 (5.8% of all calves born) were exported and there were 42,441 (2.2% of all calves born) on-farm deaths. The dataset under study detailed the 16,598 calves <56 days old which were slaughtered in Irish slaughter plants in this same period (Table 1). This was considerably lower than the 33,575 slaughtered in 2023. Indeed, 2023 was the peak year of an increasing trend with 25,949 slaughtered in 2020, 22,705 in 2021, 29,797 in 2022. In the current study, male calves (beef and dairy combined) represented 82.8% of all calves slaughtered. At 76.8%, dairy was the main type with 91.7% of those being male dairy type calves. The main breeds (>98%) represented were Friesian, Jersey and their crosses.

**Table 1:** Calves sent for early calf slaughter, calf-level descriptive statistics.

Calf level	Total	Dairy	Beef	Moved	% male^+^
Total number of calves	16,598	12,744	3,854	5,127	90.00%
Males	13,742	11,682	2,060		82.80%
Females	2,856	1,062	1794		
Majority breed type					
fr/frx	9,893			3,905	95.40%
je/jex	2,624			463	94.00%
other	4,081			759	60.00%
SDAS status					
Birth herd					
Members of SDAS	15,428	12,142	3,286	4,961	90.00%
Not members of SDAS	1,170	602	568	166	82.50%
Presenting herd					
Members of the SDAS	10,865	8,111	2,754		
Not members of SDAS	5,733	4,633	1,100		
TB status of birth herd					
Calves from TBF* BH	9,066	7,574	1,492		
Calves from TBSW** BH	7,532	5,170	2,362		
Male calves from TBF BH	7,885	7,045	840		
Female calves from TBF BH	1,180	529	653		

BH: birth herd.

+ % male: the percentage of those animals which were moved from a birth herd to a presenting herd that were male.

*TBF: bTB status of the herd free.

**TBSW: bTB status of herd either suspended or withdrawn.

A detailed analysis of the characteristics relating to the animal and herd factors related to the 16,598 calves slaughtered in 2024 is presented below. Descriptive data is presented in Tables 1–3.

A total of 5,127 calves (30.9% of all calves) were moved from the birth herd to another herd which subsequently presented the calf for slaughter. The majority of these calves (96.8%) were being moved off farms which had SDAS membership. The number of slaughtered calves born on farms which were members of the SDAS was 15,428 equating to 93.0% of all slaughtered calves, whereas the number of calves presenting for slaughter from a SDAS member farm was only 10,856 representing 65.4% of all slaughtered calves, in all cases the majority type was dairy (78.7% birth herd, 74.7% presenting herd).

Previous work has revealed that a bTB breakdown in the herd in the year of presentation for slaughter was associated with a small increase in the slaughter rate (8). The national herd incidence of bTB at the end of Q2 in 2024 was 5.2% (12-month rolling end of June 2024 herd breakdowns relative to herds tested: 5269/102182), a sizable rise from 4.6% (herd breakdowns: 4773/103400) at the same point in 2023. This rise is reflected in the current dataset as only 54.6% of the calves slaughtered in January to May 2024 were born into a bTB free herd whereas 88.1% of slaughtered calves from 2018–2022 were born into a bTB free herd.

Of the 9,066 calves born into a bTB free herd in January to May 2024, 7,574 (83.5%) were dairy and 7,045 (93.0%) of those were male. Comparing beef versus dairy herds, 60.3% of all male dairy calves presented came from a bTB free birth herd whereas only 40.8% of the male beef calves originated from birth herds that did not experience bTB. A small number of slaughtered calves (*n* = 529) were female dairy calves, born into a herd which did not experience a bTB breakdown.

A total of 1,266 herds in the dataset sent calves to slaughter (Table 2). There were 1,241 birth herds, 97.2% (*n* = 1,206) of which had SDAS membership. In contrast, only 247 herds presented calves for slaughter, and of these, 85.8% (*n* = 212) had SDAS membership. The mean number of calves slaughtered per birth herd was 13.4 calves (median: 3, range: 1–327) whereas the mean number slaughtered per herd of presentation was 67.2 (median: 23, range: 1–3,358). The majority of birth herds of the slaughtered calves sent ≤50 (*n* = 1,155 herds or 93.07%), while 71 herds sent between 51 and 200 calves. Only 13 birth herds sent > 200 calves to slaughter and the maximum sent by any one herd was 327 calves. Conversely, 172 presenting herds (69.6%) slaughtered ≤50 calves, a further 59 herds slaughtered between 50 and 200 calves and 16 herds slaughtered >200 calves in the study timeframe. In contrast to the birth herds, there was a small number of herds which slaughtered very large numbers of calves, one herd alone slaughtered 3,359 calves.

**Table 2:** Calves sent for early calf slaughter, herd-level descriptive statistics.

Herd Level	Birth Herd (count)	Presenting Herd
	Total BH	Total PH
Total number of herds	1,241	247
Calves per herd		
Mean	13.4	67.2
Median	3	23
Range	1–327	1–3358
SDAS members	1,206	212
No. of herds TBF	1,117	
Calves per herd		
Mean	8.1	
Median	2	
Range	1–256	
No. of herds TBSW	124	
Calves per herd		
Mean	60.7	
Median	29	
Range	1–327	

BH, birth herd; PH, presenting herd.

Many of the birth herds (*n* = 1,019) did not directly send any calves to slaughter but those that did (*n* = 222 herds) sent larger numbers of calves (mean 57.5, median 31, range 1–327). The average number of calves slaughtered originating from birth herds which did not themselves send the calves to slaughter was much lower (mean 3.8, median 2, range 1–98). There were 25 herds presenting calves for slaughter that were not the birth herd of the calf. These herds sent the largest number of calves for slaughter (mean 205, median 23, range 1–3,359).

At herd level, 1,117 (90.0%) of the birth herds did not experience a bTB breakdown and the mean number of calves sent by bTB free herds was 8.1 (median 2, range 1–256; Table 2). Conversely the 124 (10.0%) birth herds which had their bTB status either suspended or withdrawn had an average of 60.7 calves slaughtered (median 29, range 1–327).

Population level data is also presented in Table 3, summarizing information for the present study relative to 2023 and 2018–2022. Importantly, there was a reduction from the 2023 slaughter rate, where the whole year ECS was 1.4% for 6-week-olds, relative to 0.81% for the 2024 calving season. The slaughter rate of 0.86% in our data set included calves ≤8 weeks old. Previous work on 2018–2022 reported on average of 1.09% of live calves were slaughtered per annum, albeit including a small number of calves up to 6 months of age. Male calves continue to represent the majority, although the absolute numbers slaughtered are trending downwards.

**Table 3:** Calves sent for early calf slaughter, herd-level descriptive statistics.

Population level	2024 (Jan–May)	2023-full yr	Jan 2018–May 2022
Calves registered	1,937,533	2,398,418	11,530,000
Male calves	947,748 (49.0%)	1,196,278 (49.9%)	
Male dairy calves	271,245 (13.8%)	352,492 (14.8%)	
Calves slaughtered	16,598	33,575	1,25,260
	0.86%~	1.4% ~ ~	1.09% ~ ~ ~
	0.81% ~ ~		
Male calves	13,742 (82.8%)	30,140 (89.8%)	118,756 (94.8%)

~calves <8 weeks old.

~ ~ calves <6 weeks old.

~ ~ ~calves <6 months old.

### 3.1 Model outcome

**Figure 1:**
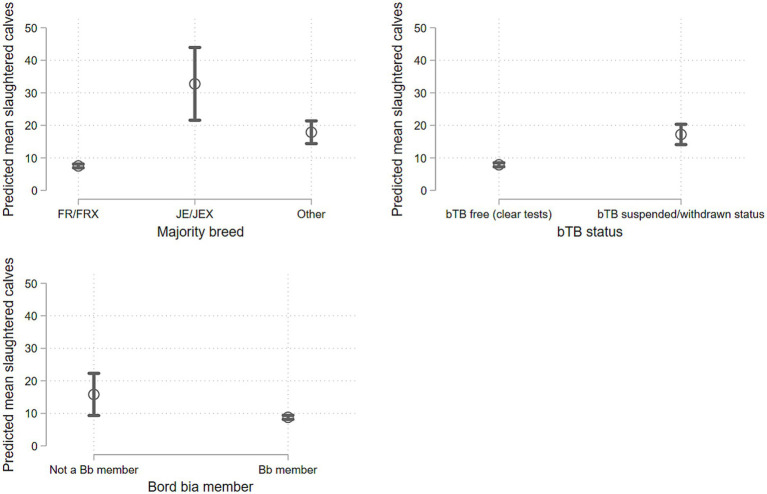
Predictions from a negative binomial count model predicting the mean calves slaughtered by breed, bovine TB (bTB) status and Bord Bia membership. bTB free = received clear tuberculin tests, and therefore legally free to trade; bTB suspended/withdrawn = bTB is suspected/bTB is confirmed or reactors are disclosed at an official test, and movement restrictions are imposed. FR/FRX, Friesian/Friesian-cross; JE/JEX, Jersey/Jersey-Cross. Bb, Bord Bia, state food agency who oversees a Sustainable Dairy Quality Assurance scheme.

Overall, there was evidence that the model significantly explained variation in counts (*p* < 0.001), and that the negative binomial model fitted the data better than a Poisson model, given the greater variance in the counts relative to the means. Mean herd size of the birth herd was a positive predictor of increasing ECS numbers (IRR: 1.003; *p* < 0.001; Supplementary Table S1; Supplementary Figure S1). Furthermore, there was evidence to suggest that birth herds that culled predominantly Jersey (IRR: 4.153; *p* < 0.001) or other breeds (IRR: 2.396; *p* < 0.001) tended to have higher ECS relative to herds that predominantly slaughtered Friesian animals (Figure 1). Herds which experienced a bTB event (i.e., a suspended or withdraw status) during 2024 tended to have higher ECS counts than herds without a bTB status (IRR: 2.031; *p* < 0.001; Figure 1).

## 4 Discussion

Our findings show that the policy intervention applied at the start of 2024 (an industry-led change to the conditions for membership of the SDAS) had a major impact on the number of calves slaughtered in the following months. This demonstrates the success of the chosen approach in influencing the management choices made by dairy farmers and is a useful example for other challenges of how stakeholder engagement and industry-centered solutions can yield change. At the same time, the policy intervention also drove some other changes in behavior which had not been sought, such as the movement of calves from the birth herd to another herd and thence to slaughter, presumably in an attempt to evade the association of the birth herd with the act of early calf slaughter. In this paper, we explored the animal and herd level factors associated with these changes and highlighted both the overall positive effect of this successful industry-led policy intervention, and also the unintended negative consequences.

Conceiving ethical and economically viable business models for the male dairy calf has persisted as an unresolved challenge for the dairy industry worldwide (1, 2, 11). In Ireland, there is no local market for veal and so the major alternatives to ECS remain at improving the CBV of the calves, finishing as a beef animal locally, exporting the calf to foreign markets or the use of sexed semen (SS) to reduce the number of male dairy calves conceived (5, 12). While none of these options are without flaws, the slaughtering of otherwise healthy young animals as a waste product is widely considered unethical and demands a more acceptable solution (13, 14). Implementation of the changes in farming practices required to reduce early calf slaughter remains largely at the discretion of the primary producers and so ‘buy in’ from these key stakeholders is paramount to the success of any strategies. This responsibility is broadly recognized by dairy and beef farmers, and a willingness to adopt some of the alternatives has been previously reported (5, 12).

Arguably the most promising of the proposed solutions is the use of SS to increase the percentage of female calves born, for use as replacement heifers either within the birth herd or sold on (15). Aside from the higher cost, there are however a number of practical barriers to using SS such as lower conception rates (92%) relative to conventional semen (16). In addition, the fertility of the recipient needs to be optimal as does the timing of Artificial Insemination (AI). SS is not a viable option for all prospective dams for all farming models. Furthermore, the contribution of SS to reducing ECS will be redundant if the number of female dairy calves born exceeds the number required to achieve the recommended 18%–20% replacement rate. Despite these challenges the use of SS has increased in Ireland with a reported 56% increase in usage in 2023 relative to 2022 (17). Selective use of dairy sired SS on the cows with the most dairy specific genetics (e.g., higher milk yield) to breed the female replacements, and using beef sires on the remainder of the herd has been suggested and may represent a practical strategy to reduce production of those male calves with the lowest CBV (5).

Notwithstanding the ongoing efforts to develop alternative uses for the male dairy calf, in January 2024 the SDAS introduced the ban on ECS as described above. Stipulated is the prohibition of intentional off-farm movement for the purpose of slaughter. However, once an animal has departed from its birth herd that herdowner has no further jurisdiction and arguably, responsibility for the outcome of that animal. Consequently, sanctioning a herd owner for early slaughter of an animal which had been sold on might prove difficult to enforce.

This study explored the different pathways from birth to slaughter across herd types, calf sex, herd affiliations and herd disease status. Considering the newly implemented industry code of practice on ECS, this study aim was to identify any emerging patterns in calf movements to slaughter and suggest possible explanations or motivations for the same.

It is clear from the data that the male dairy calf continues to represent the vast majority of calves being presented for early slaughter but encouragingly the absolute numbers of calves being slaughtered has decreased. When viewed in light of the reported increase in bTB incidence (18), a factor shown to have an association with increased calf slaughter rates, then this decrease in calf slaughter numbers in 2024 may be even more promising, likely the success of the policy intervention in its first months of operation. Indeed, while outside the time period of our study, it is of relevance to note that in the period 1st January to 7th May 2025 only 2,042 calves aged 0–8 weeks of age were reported to have been slaughtered (E. Ryan, personal communication) indicating a further reduction on the 2024 numbers.

Most farms which sent calves to slaughter sent a small number. Many of the birth farms with very low slaughter numbers did not themselves send the calves to slaughter but rather moved them to a herd which ultimately presented the calf for slaughter. This resulted in a few farms sending disproportionately large numbers of calves. One presenting herd was associated with 20% of all slaughtered calves (*n* = 3,359), with calves coming from 970 different birth herds. The average birth herd trading with this herd sent 3.5 calves that went on to be slaughtered (median: 2; IQR: 1–4; MAX: 65) and the vast majority (98%) were SDAS members. We consider this practice to be an attempt to still send the calves to slaughter but “laundering” them via another herd, with the goal of preventing an association between the birth herd and the practice of calf slaughter, and thus to frustrate attempts to enforce the new policy. Prior to the new policy, it had been rare for calves to be sent first to a new herd and thence to slaughter (8); the majority of slaughtered calves had been sent directly to slaughter up to and including 2023.

This practice of sending calves indirectly to slaughter poses two problems. Firstly, it makes enforcement of the new policy regarding SDAS membership and calf slaughter difficult. Secondly, it represents a potential dis-improvement in the welfare of these calves, which is a serious unintended consequence of the policy intervention. All else being equal, the welfare of these very young calves would be better protected by being sent directly from their birth herd to slaughter, rather than being transported to another herd for a short time and then transported to slaughter. The welfare of the calves thus suffers in order to facilitate the owner of the birth herd attempting to evade responsibility for their choices regarding calf slaughter. These findings highlight the challenges in policy development and the need to evaluate the policy impacts and iteratively review and refine policies to address unintended consequences while still achieving the overall policy goal.

One peculiar finding was the small number, 3.2% of all calves slaughtered, which were dairy type female calves from bTB free herds. These calves should have a real monetary value as replacement heifers either within the birth herd or sold on, that they would be slaughtered seems counterintuitive. The sex of the calf is as recorded and submitted by the herd owner at the time of registering the birth. One plausible explanation for this seemingly spurious finding is that the sex was misclassified and that some proportion of the 529 female dairy calves were in fact male.

In conclusion, there has been a welcome industry wide shift away from the early slaughter of calves in the dairy sector in Ireland. Overall, there has been a reduction in ECS in absolute and relative terms in comparison with prior to the SDAS prohibition. Furthermore, in comparison to preceding years, more ECS were attributable to challenges faced within herds due to on-going or new herd bTB breakdowns. However, some patterns in the data suggest that some ECS may be associated with moving animals off-farm, posing a risk to calf welfare and a challenge to policy enforcement. The paper highlights the importance of on-going analysis of near real-time data for scientific support and policy monitoring.

## Data Availability

The data analyzed in this study is subject to the following licenses/restrictions: Statistical data pertaining to calf slaughter are published and disseminated annually online by the Department of Agriculture Food and the Marine https://www.gov.ie/en/publication/467e3-cattle-aim/#aim-bovine-statistics-annualreports; data available upon request due to restrictions. Requests to access these datasets should be directed to animalwelfare@agriculture.gov.ie.
