# Supplementary material for: Early calf slaughter: impact of industry-led policy interventions on trends in Ireland, 2024
Source: Front Vet Sci. 2025 Nov 26;12:1629858. doi: 10.3389/fvets.2025.1629858 (PMC12690647; doi:10.3389/fvets.2025.1629858)

## Supplementary material:

**Table S1.** Table from a negative binomial regression model associating the number of early calf slaughters per herd in 2024 with birth herd size, breed group, bovine TB status, and Bord Bia membership. IRR = Incident Rate Ratio (exponentiated β coefficient).

| Slaughter counts per herd | IRR | Std. Err. | z | P>z | lower 95%CI | upper 95%CI |
| --- | --- | --- | --- | --- | --- | --- |
| Herd size |  |  |  |  |  |  |
| Mean herd size | 1.003 | <0.001 | 10.910 | <0.001 | 1.002 | 1.003 |
| Majority breed |  |  |  |  |  |  |
| FR/FRX  JE/JEX  OTHER | 1.000  4.359  2.379 | 0.780  0.257 | 8.230  8.030 | <0.001  <0.001 | 3.070  1.925 | 6.190  2.940 |
| bTB status 2024 |  |  |  |  |  |  |
| bTB free status  bTB suspended/withdrawn | 1.000  2.187 | 0.224 | 7.630 | <0.001 | 1.789 | 2.673 |
| QA scheme membership |  |  |  |  |  |  |
| No  Yes | 1.000  0.555 | 0.118 | -2.770 | 0.006 | 0.366 | 0.842 |
|  |  |  |  |  |  |  |
| Constant | 7.350 | 1.566 | 9.360 | <0.001 | 4.841 | 11.158 |
|  |  |  |  |  |  |  |
| alpha (overdispersion parameter)* | 1.475 | 0.056 |  |  | 1.370 | 1.589 |

* Alpha parameter is a measure of dispersion of the counts modelled, if the dispersion parameter CIs straddled zero, the data would be better fit a Poisson model.

**Figure S1:** The relationship between the number of early calf slaughters and birth herd size, based on the mean marginal prediction from the multivariable negative binomial model presented in Table S1.

**Figure S2: Directed Acyclic Graph (DAG) of the relationships between outcome and explanatory variables explored in this study. This graph demonstrates the theoretical relationships between the outcome and independent variables. Solid line represents known relationships with a strong evidence base, and we included a dotted line between breed and bTB as the evidence base is less certain given that most animals were dairy breeds and because it is conditional on exposure.**


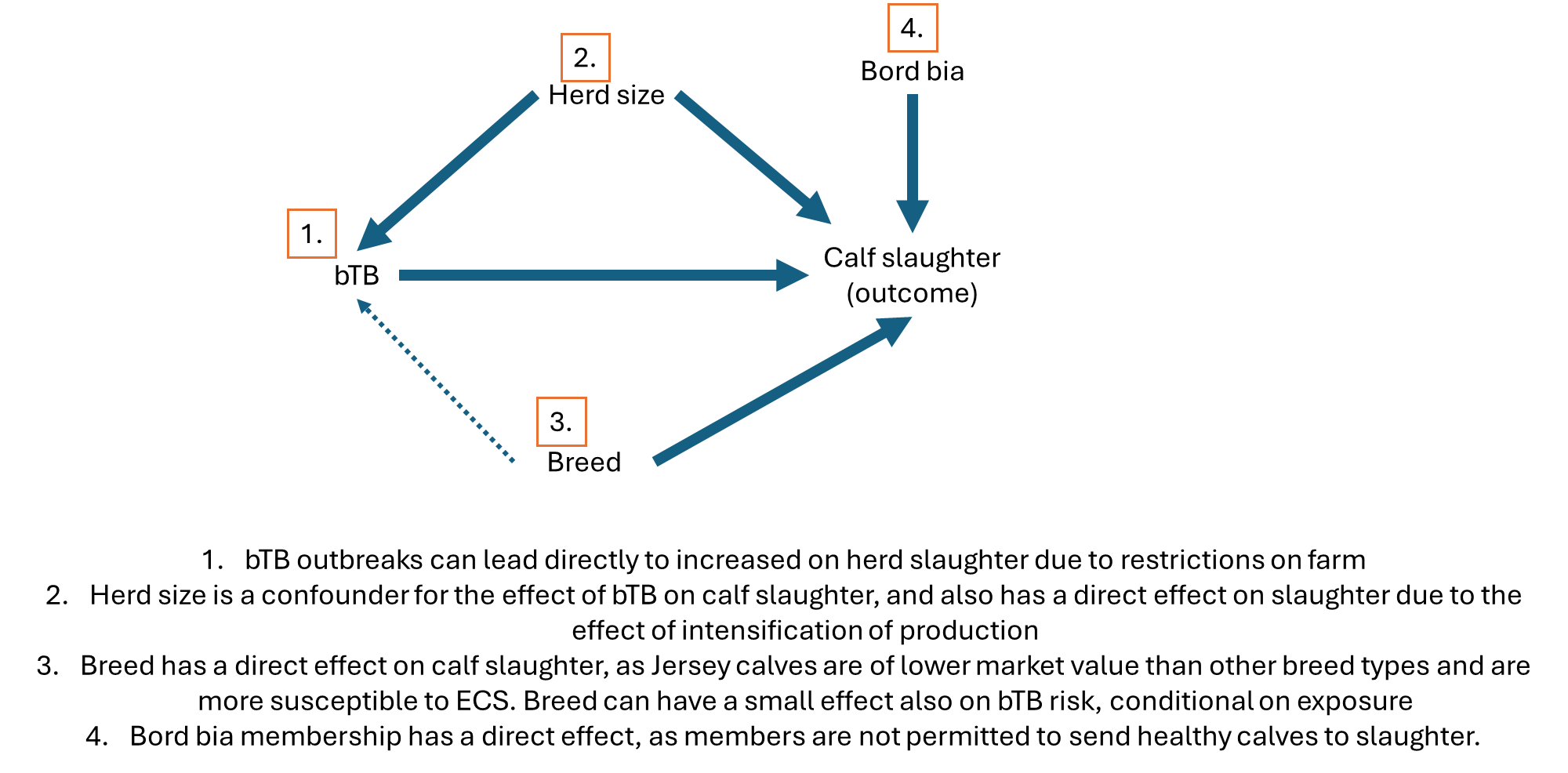

Supplement: Supplementary file 1 [file Data_Sheet_1.docx]
